# Feeding and eating problems in children and adolescents with autism: A scoping review

**DOI:** 10.1177/1362361321995631

**Published:** 2021-03-02

**Authors:** Jessica Baraskewich, Kristin M von Ranson, Adam McCrimmon, Carly A McMorris

**Affiliations:** 1 University of Calgary, Canada; 2 Alberta Children’s Hospital Research Institute, Canada

**Keywords:** autism spectrum disorders, children and youth, eating disorders, feeding disorders

## Abstract

Feeding problems, such as picky eating and food avoidance, are common in youth with autism. Other feeding and eating problems (e.g. disordered eating, fear of trying new foods, and insistence on specific food presentation) are also common in this population. This scoping review describes the nature and extent of feeding and eating problems in autistic youth and reports characteristics of autistic youth who experience such issues. Thirty-four studies were included in the current review, with almost all studies (91%) investigating feeding problems. Only 9% of studies examined concern with weight, shape, and/or body image, but several authors noted that disordered eating attitudes and behaviors may occur more frequently in those with autism than their peers without autism. No common individual characteristics (e.g. cognitive functioning and autism symptom severity) were identified for youth who experience feeding or eating problems. Although differentiating “feeding” from “eating” problems is critical for accurate identification and treatment of these issues, the existing literature has failed to do so. We propose that in future research “eating problems” be used when behaviors involve preoccupation with food, eating, or body image, and “feeding problems” be used when such preoccupation is absent.

**Lay abstract:**

Feeding problems, such as picky eating and food avoidance, are common in youth with autism. Other, broader difficulties with feeding and eating (eating disorder symptoms such as restricting food intake or preoccupation with body shape or weight and insistence on specific food presentation) are also common in autistic individuals. Here, we describe the nature and extent of feeding and eating problems in youth with autism. We found no common characteristics (such as severity of autism symptoms) that best describe autistic youth who experience problems with feeding or eating. Almost all studies we reviewed focused on problems with feeding (selective or picky eating), and only a few studies focused on eating disorder symptoms (concern with weight, shape, and/or body image). However, some researchers reported that eating disorder symptoms may occur more often in autistic individuals compared to their peers without autism. Many studies used the terms “feeding” and “eating” problems interchangeably, but understanding the difference between these problems is important for researchers to be consistent, as well as for proper identification and treatment. We suggest future researchers use “eating problems” when behaviors involve preoccupation with food, eating, or body image, and “feeding problems” when this preoccupation is absent. We highlight the importance of understanding whether feeding or eating problems are separate from autism traits, and the role of caregivers and other adults in the child’s treatment. Considerations for health-care providers to assist with diagnosis and treatment are also provided.

Autism^1^ is a neurodevelopmental condition characterized by social communication deficits and restricted repetitive patterns of behavior or interests (American Psychiatric Association [APA], 2013). Feeding and eating problems are pervasive problems that affect persons with autism across all ages and cognitive abilities (Råstam, 2008; Vissoker et al., 2015). For example, in their sample of 1462 youth, Mayes and Zickgraf (2019) found atypical eating behaviors (e.g. limited food preferences and brand-specific preferences) occur much more often in autistic children (70.4%) compared to children with other disorders (13.1%) and children in the general population (4.8%). When issues such as mealtime behaviors, fear of trying new foods, and eating problems associated with medical disorders (e.g. gastrointestinal disorders) are considered, rates of eating and feeding problems in autistic youth are likely even higher. Such high rates of varied eating and feeding problems suggest the difficulties related to eating that autistic youth experience may be complex, pervasive, and heterogeneous in nature.

Despite the heterogeneity of these problems, the most recent version of *Diagnostic and Statistical Manual of Mental Disorders* (5th ed.; DSM-5; APA, 2013) combined problems related to feeding and eating into one comprehensive chapter, highlighting similarities between the two types of disorders. Eating disorders involve persistent disturbances in eating or eating-related behaviors that significantly impair health or functioning, and often involve overconcern with weight, shape, and/or body image disturbance (APA, 2013). To date, no formal definition for feeding disorders has been established (Kennedy et al., 2018); however, a commonly accepted definition is “Severe disruptions in nutritional and caloric intake exceeding ordinary variations in hunger, food preference, and/or interest in eating” (Sharp et al., 2017, p. 116).

This definition assumes that the disturbance in eating is unrelated to concerns with weight, shape, or appearance in feeding disorders. Although abnormal feeding or eating behaviors are symptoms of feeding and eating disorders (Claudino et al., 2019), a key difference between the two categories of disorders involves the individual’s cognitive appraisal of their appearance or body image concerns. Specifically, eating disorders involve varying degrees of preoccupation with food, body weight, and/or shape, whereas in feeding disorders, the motivation may be a combination of other reasons (e.g. negative previous feeding experiences, pain/discomfort with feeding, and low muscle tone; Claudino et al., 2019; Kennedy et al., 2018) rather than cognitive concerns related to the effects of food on appearance or body image concerns.

## Eating and feeding disorders

Anorexia nervosa (AN) is an increasingly common eating disorder in which individuals limit their food intake, have a marked fear of gaining weight, and their body weight and/or shape excessively influences their self-evaluation (APA, 2013). Another eating disorder associated with concern over weight/shape is bulimia nervosa (BN), which has core symptoms of repeated episodes of binge eating (i.e. eating in a 2-h period a distinctly larger amount of food than what most individuals would eat in a similar time period and context, together with a sense of loss of control over eating) followed by purging or inappropriate compensatory behaviors to prevent weight gain (e.g. self-induced vomiting, laxative misuse, and excessive exercise; APA, 2013). A third, common eating disorder is binge eating disorder (BED), which has core diagnostic features of repeated episodes of binge eating accompanied by feelings of distress (APA, 2013).

A common eating concern that most children experience at some point during childhood is picky eating (Mascola et al., 2010). There is little consensus on precisely what defines picky eating, but the most commonly accepted definition describes a reluctance to eat familiar foods or try new foods, which interferes with daily functioning and adversely impacts the child and their caregivers, as well as the parent–child relationship (Lumeng, 2005). Picky eating is often used colloquially to refer to selective food intake (Kral et al., 2015), or eating an inadequate variety of foods that can occur for many reasons, such as sensory sensitivity (based on food texture or color, for example), limited food preferences, or neophobia (fear of trying new foods). Although picky eating and selective eating are not formal diagnoses, the DSM-5 (APA, 2013) introduced a disorder that resembles a persistent, extreme version of picky eating, avoidant/restrictive food intake disorder (ARFID). This disorder is characterized by avoidance of certain foods, resulting in a limited repertoire of foods an individual will eat, and/or restricted food intake leading to nutritional or energy deficits (APA, 2013). Whether ARFID best represents an eating disorder or a feeding disorder has been contested, as symptoms share characteristics with both. For example, similar to feeding disorders, ARFID can only be diagnosed in the absence of weight or shape concerns; however, symptoms such as rigid rules around food and low weight mirror what is commonly seen in AN, highlighting its similarity to eating disorders (Kennedy et al., 2018).

Finally, the DSM-5 (APA, 2013) outlines two feeding disorders, pica and rumination disorder, about which little is known, but both disorders are thought to be over-represented in persons with autism (Chial et al., 2003; Matson et al., 2011; Råstam, 2008). Pica is characterized by persistent eating of non-nutritive, non-food substances that is not part of cultural or social normative practices (APA, 2013). A diagnosis of rumination disorder applies when an individual repeatedly regurgitates food (i.e. food is swallowed and then brought up into the mouth and either re-chewed and ejected or re-swallowed), without attribution to an associated gastrointestinal or other medical condition (APA, 2013).

## Feeding and eating problems in persons with autism

Research on eating behaviors in persons with autism is relatively limited (Råstam, 2008), and existing literature has focused on a broad range of difficulties, including feeding problems, mealtime behaviors, and picky eating. Most literature to date examining eating disorders in persons with autism has been conducted predominately with adults, whereas picky eating and feeding disorders have been investigated in children and youth. Feeding or eating problems commonly present early in development; often even before concerns related to autism are identified (Emond et al., 2010). Without treatment, the feeding problems autistic children experience tend to persist into late childhood (Suarez et al., 2014); however, to our knowledge, no studies have examined whether these feeding problems precede eating problems in autistic youth.

Recently, increased attention has been directed at the overlap of autism and eating disorders, with particular emphasis on AN (e.g. Karjalainen et al., 2019; Kinnaird et al., 2019; Westwood & Tchanturia, 2017). Westwood and colleagues (2018) found an overrepresentation of autism symptoms in adolescent females with severe AN, with 10% of their sample meeting full criteria for autism and an additional 40% who were below cut-off but presented with symptoms. Most literature to date has examined the presence of autism symptoms in individuals with AN, and a few studies have examined the reverse relation.

Having a neurodevelopmental condition such as autism may increase the likelihood of developing eating problems, although this relation is poorly understood (Mayes et al., 2018). Several mechanisms may explain this association. For example, cognitive inflexibility might manifest as rigid rules around food and a preoccupation with eating that may develop into disordered eating with similar characteristics of rigidity and an obsession with food/eating such as AN (APA, 2013). Alternatively, there may be a shared underlying genetic vulnerability that interacts with environmental factors to manifest as disordered eating, or shared underlying difficulties in cognitive, social, and/or emotional functioning (Davies et al., 2016; Oldershaw et al., 2011; Westwood et al., 2016). Recently, Brede et al. (2020) proposed a model of autism-specific traits that may influence the development and maintenance of restrictive eating problems. These traits included sensory sensitivities, social interaction and relationship difficulties, sense of self and identity issues, difficulties with emotions, autistic thinking styles, and a need for control and predictability that may interact to influence a variety of restrictive eating presentations directly and indirectly.

Based on the field’s limited understanding of eating problems in autism, eating problems are likely often misattributed to the individual’s autism traits (Mayes et al., 2018). For example, the restriction of food intake, a primary symptom of AN, can be misattributed as a sensory aversion. Although eating problems may be secondary to an autism diagnosis, no consensus has been established for how to assess for eating problems in this population. Similarly, there is a limited understanding as to whether such problems should be attributed to an individual’s autism or whether an additional diagnosis is warranted. Likewise, psychotherapeutic treatment options that adequately address an autistic person’s eating problems are extremely limited.

## Current review

The purpose of this review is to comprehensively describe the nature and extent of feeding and eating problems in youth with autism. The aims of this article are to: (1) summarize commonly investigated feeding and eating problems as well as quantify the percentage of studies that assess weight, shape, and/or body image concerns in youth with autism and (2) identify characteristics that may influence the prevalence and/or presentation of feeding and eating problems. Implications for diagnosis and treatment of feeding and eating problems in autistic individuals are discussed.

## Methods

### Search strategy and selection criteria

**Figure 1.**
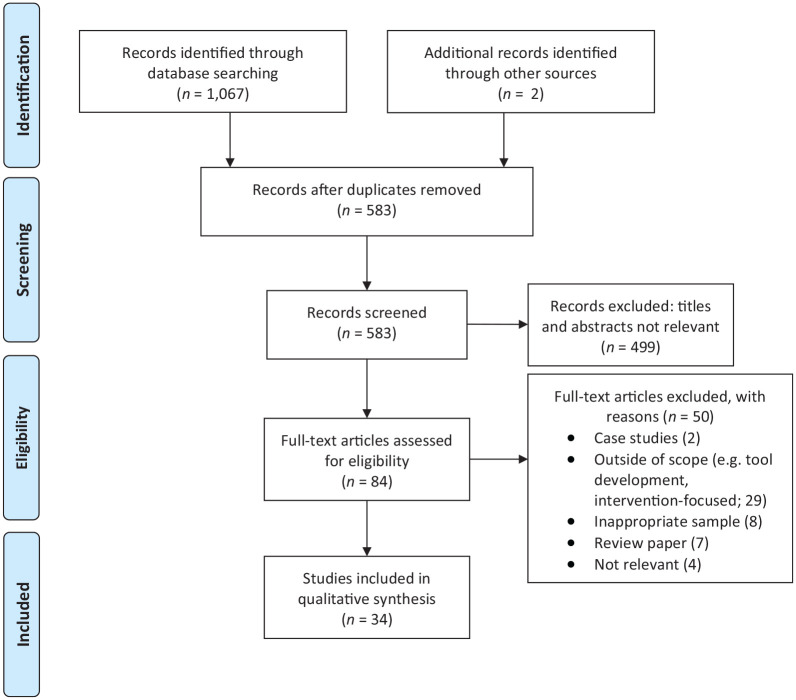
PRISMA diagram results (from Moher et al., 2009).

A systematic literature search was conducted in MEDLINE, PsycINFO, and PubMed databases using relevant controlled vocabulary and key terms (see Table 1). The review was conducted and reported according to the Preferred Reporting Items for Systematic Reviews and Meta-Analyses (PRISMA; Moher et al., 2009), as shown in Figure 1. Publication dates were not restricted to ensure all possibly relevant articles were included up until April 2020. All searches were restricted to articles written in English, conducted with human participants, and published in peer-reviewed journals. The systematic search was accompanied by a manual examination of reference lists from retained articles.

**Table 1.** Search terms.

Population terms	autism spectrum disorders OR pervasive developmental disorder OR PDD^a^ OR asperg^a^ OR autis^a^
Age terms	child^a^ OR infant^a^ OR adolescen^a^ OR pediatr^a^ OR youth^a^
Feeding and eating problems terms	Feeding and eating disorders OR feeding behaviour OR ‘eating issue’ OR ‘eating problem’ OR ‘picky eating’ OR eating behavior^a^ OR eating disorders or body image OR eating attitudes OR food neophobia OR food preferences OR food intake

PDD: pervasive developmental disorder.

a Denotes wildcard search terms.

To be included, articles had to contain empirical research with a focus on feeding and/or eating problems, not exclusively medical-related issues (e.g. studies primarily focusing on nutrient deficiencies, genetic markers, and parental feeding practices). To meet these criteria, articles had to include an objective measure of feeding or eating problems. Studies were also required to analyze and present data from persons with autism (or equivalent diagnoses such as pervasive developmental disorder–not otherwise specified (PDD-NOS) or Asperger’s syndrome) separately. Samples had to focus on children or adolescents aged 18 years or under; however, in line with previous reviews of mental health issues in persons with autism (e.g. Cassidy et al., 2018), studies were included if 50% or more of the total sample was under 18 years, to maximize the number included. Studies that examined feeding and eating problems in the context of other medical conditions (e.g. gastrointestinal disorders and epilepsy) were excluded to provide a homogeneous evidence base. Studies examining interventions for specific behaviors (e.g. swallowing and number of bites accepted), case studies, and tools developed to address feeding and eating problems were also excluded to allow for better generalizability of the findings.

### Community involvement

Community service providers who specialize in treating feeding and eating disorders were consulted in the development of the broad aims of this article. After the search was completed, findings were presented to service providers and feedback from this consultation was incorporated into our discussion. Of note, while these individuals have extensive expertise in treating feeding and eating issues in autistic youth, they themselves are not autistic. Thus, their views and input reflected their professional experiences rather than lived experience.

## Results

We included 34 studies in our review, with 22 studies appearing to use original data sets and 12 reporting secondary data analysis. The total sample of participants with autism was 4215. Participants were recruited from a variety of sources (e.g. community organizations, autism service organizations, and specialized clinics). Most studies (59%) were conducted in the United States, with some completed in Australia (9%), as well as Italy, England, and Canada (6% each). The earliest study was published in 2004, and over half (56%) were published from 2013 to 2017. Participant age ranged from 2 to 28 years, although the majority focused on children 8 years of age or younger. Most studies (88%) relied on parent-reported data; a small number (9%) combined parent- and self-report, and a single study (3%) relied solely on observational data. Nearly, all studies (88%) used a cross-sectional design, although some (12%) collected data across multiple time-points. Table 2 presents sample characteristics and relevant findings of included studies.

**Table 2.** Studies of feeding and eating issues in children and adolescents with autism.

Author (year)	*N* ^a^	Sample characteristics^b^	Age range (mean, M)	Feeding/eating measure	Control group(s)^c^	Primary findings
Aponte & Romanczyk (2016)	38	AD (58%), AS (11%), PDD-NOS (18%), ASD (13%). Sex: 84% male	3–12 years (M: 6)	BAMBI, FFQ	None	ASD severity predicted feeding problem scores and duration of negative vocalizations during meal observations. 39% of participants showed low food acceptance.
Attlee et al. (2015)	23	Autism. Sex: 78% male	5–16 years (M: NA)	BAMBI, FPI, 3-day food record	None	Participants had mealtime concerns related to limited variety, food refusal, and picky eating. Sample had a high rate of food rejection.
Bandini et al. (2010)	53	Autism. Sex: 83% male	3–11 years (M: 6)	FFQ, 3-day food record	TD (58)	Autistic children had significantly more food refusal and limited food repertoire vs controls.
Bandini et al. (2017)	18	Autism. Sex: 89% male	NA (baseline M: 6; follow-up M: 13)	FFQ, MIOH, 3-day food record	None	Food refusal improved from baseline to follow-up, but repertoire of foods eaten did not. Rates of high food selectivity (FS) decreased from 83% to 44% between time-points.
Beighley et al. (2013)	269	AD (127), PDD-NOS (82), AS (60). Sex: 70% male	2–18 years (AD M: 7; PDD-NOS M: 7; AS M:10)	ASD-CC	Atyp Dev (107), TD (149)	Children with ASDs had more FS vs non-ASD groups. There was a downward trend in FS severity across childhood; as children got older FS decreased.
Bitsika & Sharpley (2018)	52	ASD. Sex: 100% female	6–17 years (M: 10)	SWEAA	None	11% of scores from parents and daughters fell into the “usually-always” range.
Castro et al. (2016)	49	ASD. Sex: 100% male	4–16 years (M: 10)	BPFAS	TD (49), matched	ASD group had higher levels of overall problematic eating behavior, but did not significantly differ from controls on factor scores (e.g. picky eating, refusal based on food texture, and general refusal).
Chistol et al. (2018)	53	Autism. Sex: 83% male	3–11 years (M: 6)	FFQ, Sensory Profile	TD (58)	Autistic children were more likely to demonstrate oral sensory sensitivity. Autistic youth with atypical oral sensory sensitivity refused more foods and demonstrated more restrictive eating behaviors.
Curtin et al. (2015)	53	Autism. Sex: 83% male	3–11 years (M: 6)	FFQ, MIOH	TD (58)	Children in autism group were more likely to have high FS and mealtime behavior problems.
Diolordi et al. (2014)	33	Autism. Sex: 82% male	2–9 years (two ranges: 2–5.9 years, 6–9.5 years)	CEBI, author-created food frequency measure	TD (35); age-matched	Autistic children showed more eating problems in 6–9.5 years age range. Autism group had more mealtime rituals and generally ate below recommended amounts of all food groups. Overall, eating problems decreased at 1-year follow-up for the autism group.
Emond et al. (2010)	79	ASD. Sex: NA	6, 15, 24, 38, and 54 months	Author-created food frequency questionnaires	TD (12,901)	Children with ASD demonstrated feeding symptoms from infancy and had less varied diet from 15 months of age.
Hubbard et al. (2014)	53	Autism. Sex: 83% male	3–11 years (M: 6)	FFQ, Sensory Profile	TD (58)	Autistic children refused significantly more foods for more reasons (e.g. taste/smell). Consistency/texture was the most common reason for both groups, but prevalence was much higher in autism (77.4% vs 36.2%)
Johnson et al. (2008)	19	Autism. Sex: NA	2–4 years (M: 39.2 months)	Author-created feeding assessment survey, FFQ, 24-h dietary recall	TD (15)	Autistic children had more feeding problems, particularly related to idiosyncratic refusal of foods based on color, texture, and type. Groups did not differ on nutritional status.
Johnson et al. (2014)	256	Autism (174), AS (21), PDD-NOS (57). Sex: 84% male	2–11 years (M: 5)	BAMBI, Short Sensory Profile, 3DFR Healthy Eating Index	None	Strong associations between feeding habits and repetitive and ritualistic features, sensory features, and externalizing and internalizing behavior. No association between feeding behaviors and social and communication deficits and IQ.
Kalyva (2009)	56	AS. Sex: 100% female	12–18 years (M: 14)	EAT-26 (self and parent report)	TD (56); age- and BMI-matched	AS group showed significantly more eating problems according to self- and mother-report.
Kerwin et al. (2005)	89	PDD-NOS (51%), AD (39%), AS (9%). Sex: 79% male	3–17 years (M: 8)	Author-created eating behavior measure, GI symptoms, related behavior	None	Majority of children had unusual eating habits. Only 6.7% of parents indicated their child had a feeding problem. 62% of children had FS, and 29% of the sample exhibited pica.
Kral et al. (2015)	25	ASD. Sex: 72% male	4–6 years (M: 5)	Modified Child Neophobia Scale, 35-item CEBQ, CFQ, PFSQ	TD (30)	Autistic children were at increased risk for food avoidance behaviors. ASD group had significantly more food fussiness than controls; those with ASD and oral sensory sensitivity showed more food avoidance, food fussiness, and emotional undereating.
Kuschner et al. (2015)	65	ASD. Sex: 89% male	12–28 years (M: 16)	AASP	TD (59)	ASD group was significantly more likely to be food neophobic, more likely to report disliking textured foods, and less likely to enjoy strong tastes vs controls. FS issues appeared to be linked to daily living skills.
Malhi et al. (2017)	63	ASD. Sex: 91% male	4–10 years (M: 6)	CEBI, 3-day food record	TD (50); age- and SES-matched	ASD group had significantly more feeding problems and ate significantly fewer food items. Autistic children were 6× more likely to be picky eaters than controls and were particularly averse to eating vegetables and fruits.
Marshall et al. (2016)	33	ASD. Sex: 76% male	2–6 years (M: 52 months)	BPFAS, 3-day prospective diet record	Non-medically complex history (35)	Children with ASD showed increased FS; differences between ASD and controls were found predominantly on measures of general behavior rather than feeding behaviors.
Martins et al. (2008)	41	AD (35), AD or PDD-NOS diagnosis but under CARS cut-off (6). Sex: 83% male	2–12 years (M: 7)	Author-created eating behavior questionnaire, BPFAS, FNS	TD (41), TD siblings of ASD sample (14); gender- and age-matched	Children with ASDs were marginally more likely to exhibit picky eating than their siblings or matched TD children. Rates of ritualistic feeding behaviors were similar across groups, but children with autism were more likely to have current problematic eating and feeding behaviors.
Matson et al. (2009)	114	Autism (72), PDD-NOS (40). Sex: 66% male	3–16 years (M: 8)	ASD-CC	TD (114), Atyp Dev (53)	No significant differences between autism and PDD-NOS group on measures of feeding problems. Significantly higher rates of feeding problems across all items in ASD group vs atypically or typically developing. ASD group showed problems with FS and eating style (pica and rapid eating).
Mayes & Zickgraf (2019)	1462	Sample 1: Autism (1443). Sex: 79% male; Sample 2: Autism (19). Sex: 51% male	Sample 1: 1–17 years (M: 6). Sample 2: 1–18 years (M: 10)	CASD	Other Disabilities (327), TD (313)	Atypical eating behaviors 5× more common in autism group than other disorder group and 15× more common than typical development group. Limited food preferences were the most common atypical eating behavior in autism group. Most children with autism had 2+ types of atypical eating (e.g. sensitivity to textures and idiosyncratic mealtime rituals).
Nadon et al. (2011)	95	Autism (61%), PDD-NOS (29%), AS (10%). Sex: 92% male	3–10 years (M: 7)	Eating Profile, Short Sensory Profile	None	Children with higher sensory problems had significantly more eating problems. Tactile sensitivity, taste and smell sensitivity, and visual/auditory sensitivities were associated with significantly more eating problems.
Peverill et al. (2019)	396	ASD. Sex: 84% male	24–60 months; data collected across four time-points	BPFAS	None	Feeding problems followed four distinct trajectories: (1) began low and remained stable; (2) moderate at outset and declined over time; (3) high during preschool which declined to average by school age; and (4) severe, chronic feeding problems. Feeding problems were more highly correlated with general behavior problems than with ASD symptom severity. Few strong predictors of feeding problems were identified.
Postorino et al. (2015)	158	ASD. Two groups: ASD + FS (79); age- and gender-matched ASD + no FS (79). Sex: 86% male	3–12 years (M: 7)	Revised FFQ, parent interview	None	92% of parents of children with FS observed food refusal in their child. All FS children showed at least one sensory factor linked to FS; 41% showed two sensory factors; and 23% showed three sensory factors. Children in FS group had significantly higher ASD symptoms and significantly lower cognitive functioning vs no FS group.
Råstam et al. (2013)	377	ASD only (89), ASD + ADHD (288). Sex: 74% male	9 and 12 years	A-TAC Feeding/Eating Module	ADHD only (903), no ASD + no ADHD (11,024)	Prevalence of eating problems was significantly higher in children with ADHD and/or ASD. Social interaction problems were strongly associated with eating problems in girls, and impulsivity and activity problems were associated with eating problems in boys.
Schreck & Williams (2006)	138	Autism (100), PDD-NOS (47), AS(29). Sex: 88% male	4–12 years (M: 8)	CEBI, FPI	None	Children ate only a small variety of presented foods. Restricted variety was primarily related to food presentation (e.g. utensils and food items touching on plate). Selectivity was not related to autism symptoms.
Schreck et al. (2004)	138	Autism. Sex: 88% male	7–9 years (M: 8)	CEBI, FPI	No autism or PDD diagnosis (298)	Autism group showed higher feeding problems, refused more foods, were more likely to require specific utensils/food presentations, and had preference for low texture foods vs controls. 72% of autistic children ate a narrow variety of foods.
Sharp et al. (2013)	30	ASD. Sex: 77% male	3–8 years (M: 68 months)	FPI, BAMBI, mealtime observation	None	In laboratory observation, FS was associated negatively with children’s acceptance of bites (i.e. food offered) and positively with mealtime behavior. Increased FS was positively correlated with problem behaviors during the observation, while ASD symptom severity was unrelated to feeding data.
Suarez et al. (2014)	52	ASD. Sex: 88% male. Follow-up sample ranging from 11 to 21 months	4–12 years (M: 8)	Author-created FS questionnaire	None	No change in FS level across time-points. There was a stable, significant relationship between FS and sensory over-responsivity; children with higher sensory sensitivities also had higher FS.
Suarez (2017)	31	ASD. Sex: 90% male	4–14 years (M: 9)	Laboratory food acceptance	TD (21)	Significant relationship between foods accepted and age for ASD group. Children with ASD accepted significantly fewer foods total and fewer foods from each group compared to controls, except snack foods. No relationship between foods accepted and ASD symptom severity.
Wallace et al. (2018)	37	ASD. Sex: 89% male	8–11 years (M: 10)	Child Neophobia Scale	Non-ASD (4564)	Children with ASD had more FN than peers. Subclinical associations found between FN and ASD traits. Higher FN was associated with lower BMI, but the combination of increased ASD traits and increased FN was linked with increased BMI.
Williams et al. (2005)	64	ASD. Sex: 91% male	24–129 months	Food frequency questionnaire, 3-day food diary	Special needs (45), TD (69)	No significant differences between groups on types of food consumed or liquid intake. Autistic children had more mealtime behaviors related to insistence on sameness (e.g. utensils and food preparation methods).

AD: autistic disorder; AS: Asperger’s syndrome; PDD-NOS: pervasive developmental disorder–not otherwise specified; ASD: autism spectrum disorder; NA: not available; GI: gastrointestinal; ADHD: attention-deficit hyperactivity disorder; BAMBI: Brief Autism Mealtime Behavior Inventory (Lukens & Linscheid, 2008); SES: socioeconomic status; FFQ: food frequency questionnaire (checklist to obtain frequency of food and beverage consumption; exact questionnaire varied by study); FPI: food preference inventory (unstandardized measure to ascertain food preferences); TD: typically developing; MIOH: Meals in Our Household Questionnaire (Anderson et al., 2012); FS: food selectivity; ASD-CC: Autism Spectrum Disorder–Comorbidity for Children (Matson & Gonzalez, 2007); Atyp Dev: atypically developing; SWEAA: Swedish Eating Assessment for Autism spectrum disorders (Karlsson et al., 2013); BPFAS: Behavioral Pediatrics Feeding Assessment Scale (Crist & Napier-Phillips, 2001); CEBI: Children’s Eating Behavior Inventory (Archer et al., 1991); 3DFR: 3-day food record (Guenther et al., 2006); IQ: intelligence quotient; EAT-26: Eating Attitudes Test 26 (Garner et al., 1982); BMI: body mass index; CEBQ: Child Eating Behavior Questionnaire (Wardle et al., 2001); CFQ: Child Feeding Questionnaire (Birch et al., 2001); PFSQ: Parental Feeding Style Questionnaire (Wardle et al., 2002); AASP: Adult/Adolescent Sensory Profile (Brown & Dunn, 2002); CARS: Childhood Autism Rating Scales (Schopler et al., 1980); FNS: Food Neophobia Scale (Pliner & Hobden, 1992); CASD: Checklist for Autism Spectrum Disorder (Mayes, 2012); A-TAC: The Autism-Tics, AD/HD and Other Comorbidities Inventory (Larson et al., 2010); FN: food neophobia.

a Size of ASD sample.

b Diagnosis: description of diagnosis. AD: autistic disorder, AS: Asperger’s syndrome, PDD-NOS: Pervasive developmental disorder–not otherwise specified, ASD: sample not characterized by specific diagnoses, ASDs: where groups of varying ASD diagnoses were combined.

c Composition of control group/comparison scores (*N* = number of subjects).

### Conceptualization of feeding and eating problems

Most often, the terms “feeding” and “eating” problems were used interchangeably to describe a number of related issues. Some studies used the term “feeding problems” to describe a range of problematic behaviors that occur in the mealtime context (e.g. Aponte & Romanczyk, 2016; Johnson et al., 2014) whereas others referred to such issues as “eating problems” (e.g. Kerwin et al., 2005). Others used the term “eating problems” to describe pathology related to restricted eating (i.e. failing to gain weight and perceived fear of gaining weight; Råstam et al., 2013). In contrast to the framework for distinguishing feeding and eating problems in this review (where the differentiating factor is the presence or absence of cognitive concerns related to weight, shape, and/or body image), no clear differentiation was presented in the literature.

### Commonly investigated feeding and eating problems

#### Weight, shape, and body image concerns

A primary objective of our review was to identify commonly investigated feeding and eating problems in youth with autism. A secondary, related objective was to understand how studies differentiated between feeding and eating problems by identifying the proportion of studies that measured the presence of eating disorder symptomology (defined as the presence of cognitive concerns related to weight, shape, and/or body image). 91% of included studies examined “feeding” rather than “eating” problems. Only three studies (9%) examined constructs that fit our definition of eating problems. Specifically, Bitsika and Sharpley (2018) found approximately 11% of 52 young females with autism endorsed severe eating disturbances (i.e. purging, dieting, and fasting behaviors). These authors concluded that eating disturbances do not represent a common comorbidity in young autistic females. In contrast, other researchers reported that eating problems are over-represented in individuals with autism compared to the general population. For example, Kalyva (2009) found adolescent females with Asperger’s syndrome were at a significantly higher risk for eating problems compared to age- and body mass index (BMI)-matched peers without Asperger’s syndrome. Specifically, participants with Asperger’s syndrome self-reported significantly more BN symptoms, food preoccupation, oral control, and overall eating problems. The same differences between groups were seen in parent-reported eating problems, but mothers of adolescents with Asperger’s syndrome also indicated their daughters had significantly more dieting behaviors. Similarly, in a large population-based study, Råstam and colleagues (2013) found the prevalence of restrictive eating problems (consistent with a diagnosis of AN) was significantly higher for children (aged 9 and 12 years) with attention-deficit hyperactivity disorder (ADHD) and/or autism, with the highest rates seen in girls with co-occurring ADHD and autism. Taken together, although the proportion of studies examining eating problems in autistic youth is small, preliminary evidence suggests persons with autism may be at higher risk for developing eating problems compared to persons without autism.

#### Feeding problems

86% of studies investigated feeding problems involving selective intake. A few studies reported no or marginal differences in food selectivity between youth with autism and controls. For example, although Castro and colleagues (2016) found autistic youth (aged 4–16 years) differed from matched general population controls on overall levels of problematic eating behavior, groups did not significantly differ in their picky eating symptoms. Similarly, autistic children aged 2–12 years were only marginally more likely to exhibit picky eating behavior when compared to age- and gender-matched siblings and children without autism (Martins et al., 2008). However, most studies reported greater food selectivity in autism groups. For instance, Mayes and Zickgraf (2019) found atypical eating behaviors (e.g. limited food preferences, texture sensitivity, and brand-specific preferences) were five times more common in youth with autism than children with other disorders (e.g. ADHD, intellectual disability, language disorder, and learning disability) and 15 times more common in the autism group than their typical developing peers, with limited food preferences being the most common atypical eating behavior. Matson et al. (2009) found autistic youth showed significantly higher rates of feeding problems compared to typically and atypically developing groups (i.e. developmental disability other than autism), especially with food selectivity. In general, most studies that compared groups of youth with autism to groups with other disorders and/or typical development found autistic individuals had higher rates of food selectivity. In the studies without comparison groups, rates of food selectivity in samples of autistic youth were strikingly high (e.g. 62% showed food selectivity in Kerwin et al., 2005; 72% showed restricted variety; and 57% showed food refusal in Schreck & Williams, 2006).

Four studies examined patterns of food selectivity across time and produced mixed results. Beighley and colleagues (2013) found a general trend of decreased food selectivity as children with autism got older, but the same trend was not observed in children and adolescents without autism. Interestingly, Bandini et al. (2017) examined food selectivity in youth with autism at two time-points approximately 6 years apart. They found that while food refusal improved over time, the number of foods eaten did not increase, suggesting that the decrease in overall selectivity may be better attributed to a decrease in caregivers offering non-preferred foods (Bandini et al., 2017). Similarly, Suarez and colleagues (2014) found no change in food selectivity in children with autism over a 2-year period. In perhaps the most comprehensive study of trajectories of feeding problems, Peverill et al. (2019) characterized feeding patterns of 396 preschoolers with autism across four time-points up to age 6 years. Four trajectories of feeding problems were found: less severe and stable (26%), moderate and declining (39%), severe and declining (27%), and very severe and stable (8%). Authors concluded that, like general population children, most feeding problems remitted over time, although a small group of preschoolers with autism continued to show chronic feeding problems into school age. Together, results of the four studies examining selective eating over time suggest variability in patterns of selective eating. The trajectory of selective eating is likely heterogeneous and complicated by other feeding/eating problems the child may be experiencing, including general mealtime behaviors and rituals around eating.

#### Selective eating and sensory sensitivity

Of the 29 studies that investigated selective eating, 69% (*n* = 20) also examined the presence of sensory sensitivities or aspects of food texture, temperature, smell, or taste in relation to food selectivity. Most (*n* = 15) examining sensory sensitivity and limited food intake utilized a broad measure of sensitivity (i.e. overall sensory impairment score or more focused but representative score, such as overall oral sensitivity). In general, these studies suggested food selectivity may be related to sensory impairments, although two (Aponte & Romanczyk, 2016; Schreck & Williams, 2006) found no relation between food selectivity and sensory impairments.

Five studies characterized specific aspects of sensory processing and produced comparable results. In survey-based studies, researchers found that food textures are the primary reason for food refusal in autistic youth (Hubbard et al., 2014; Mayes & Zickgraf, 2019; Nadon et al., 2011). Specifically, smooth creamy textures (e.g. mashed potatoes), foods that require chewing (e.g. unprocessed meat), and foods with lumps (e.g. oatmeal) were identified as problematic (Mayes & Zickgraf, 2019). Food selectivity was also linked to more than one sensory factor (Postorino et al., 2015), with taste and color identified as influential (Hubbard et al., 2014; Nadon et al., 2011; Postorino et al., 2015). Laboratory mealtime observations demonstrated similar findings. Sharp and colleagues (2013) found approximately half their sample of autistic children aged 3–8 years demonstrated selective patterns of eating by type and/or texture. Specifically, smoother, consistent textures (e.g. hotdogs) were more likely to be accepted than lumpy/inconsistent textures (e.g. pureed beans). Together, studies that employed more rigorous measures of sensory sensitivity suggest an association between sensory sensitivity and food selectivity, with food texture representing a strong contributing factor.

#### Rituals and idiosyncratic eating behaviors

Six studies support a relation between ritualistic behaviors and food selectivity. Several studies found youth with autism had more ritualistic and/or idiosyncratic eating behaviors (e.g. requiring specific presentation of food, and use of certain utensils) than youth with other disabilities (e.g. ADHD, intellectual disability, and language disorder; Mayes & Zickgraf, 2019; Williams et al., 2005) and general population peers (Diolordi et al., 2014; Mayes & Zickgraf, 2019; Williams et al., 2005). Specific presentation of food was identified repeatedly as the most common eating ritual (Diolordi et al., 2014; Schreck & Williams, 2006; Williams et al., 2005). Postorino and colleagues (2015) found nearly 13% of autistic children refused food because of brand or packaging, while nearly 4% had unspecified eating rituals. Although Martins et al. (2008) found no difference in eating rituals between autistic children and their siblings without autism, they found greater sensory impairment was associated with more ritualistic eating behaviors which, as outlined above, have strong associations with food selectivity.

#### Pica

Four studies examined symptoms of pica in youth with autism. Emond and colleagues (2010) found autistic children were markedly more likely than controls to show pica behavior at 38 and 54 months old. Similar results were found in an older sample, where those with autism had significantly higher rates of eating unspecified non-food items (Matson et al., 2009). Mayes and Zickgraf (2019) also found relatively high rates of pica, with nearly 12% of their large sample of autistic youth showing pica symptoms versus none in comparison groups. Finally, Kerwin and colleagues (2005) found almost 30% of their sample of autistic children had symptoms of pica. While results suggest pica may occur at higher rates in youth with autism compared to their peers, it should be noted that most of these studies did not use standardized measures to assess for symptoms of pica. For instance, two studies (Emond et al., 2010; Kerwin et al., 2005) used author-created measures to assess feeding behaviors while Mayes and Zickgraf (2019) used a validated measure along with additional qualitative information to characterize the feeding problems (e.g. inedible substances consumed).

### Characteristics of persons with autism and feeding or eating problems

A second objective of this review was to identify characteristics that may influence the prevalence and/or presentation of feeding and eating problems. Specifically, we sought to identify clinical characteristics, including cognitive functioning (intelligence quotient (IQ)), adaptive functioning, and autism traits, to understand factors associated with the presence of feeding and eating problems in autism.

#### Cognitive functioning

Although most studies included a measure of cognitive functioning within their protocols, only four studies examined the relation between feeding/eating problems and cognitive functioning. Of these four studies, just one (Postorino et al., 2015) reported a significant association between IQ and feeding problems, where lower IQ was related to higher levels of food selectivity. Conversely, Mayes and Zickgraf (2019) found that autistic youth with and without an intellectual disability did not differ in their rates of atypical eating behaviors. Similarly, others found no differences in feeding or eating problems based on IQ (Bitsika & Sharpley, 2018; Johnson et al., 2014). Of note, only Bitsika and Sharpley (2018) restricted their sample to adolescents with higher cognitive abilities (IQ ⩾70), although Johnson et al. (2014) reported 63% of their sample had an IQ of ⩾70 and Mayes and Zickgraf (2019) reported 70% of their sample had an IQ of ⩾80.

#### Adaptive functioning

Similarly, mixed results were found in the studies examining the relation between feeding and eating problems and adaptive functioning (*n* = 4). Kuschner and colleagues (2015) found that in a sample of adolescents and young adults with autism, those with food neophobia received significantly lower parent ratings of daily living skills, but scores of social and communication skills did not significantly differ from their peers with autism and without food neophobia. Conversely, the remaining three studies had younger participants (aged 2–12 years) and found no significant relation between feeding and eating problems and measures of adaptive functioning (Martins et al., 2008; Peverill et al., 2019; Postorino et al., 2015).

#### Autism traits

Finally, one-third of studies (*n* = 12) examined feeding or eating problems and autism traits, with split results. In three studies, authors used the comparison score of the Autism Diagnostic Observation Schedule–Second Edition (ADOS-2) as a measure of autism symptom severity and found no significant associations between feeding or eating behaviors and more autism symptoms (Bitsika & Sharpley, 2018; Johnson et al., 2014; Peverill et al., 2019). Likewise, three other studies found autism symptom severity was not significantly related to feeding or eating problems (Schreck & Williams, 2006; Sharp et al., 2013; Suarez, 2017). However, Postorino and colleagues (2015) used several measures to assess autism symptom severity and found those with food selectivity scored significantly higher on the Social Responsiveness Scale (Constantino, 2005) and Social Communication Questionnaire (Rutter et al., 2003). Findings from several other studies also provided support that higher overall scores on measures of autism symptoms (Aponte & Romanczyk, 2016; Schreck et al., 2004; Wallace et al., 2018), as well as specific autism traits such as difficulty adapting to change (Martins et al., 2008) and restrictive and repetitive behaviors (Suarez et al., 2014), are significantly associated with higher levels of feeding and eating problems.

## Discussion

Although the relation of autism to feeding and eating problems is not fully understood, extant literature suggests that such problems affect a substantial number of persons with autism. Consistent with previous reports (e.g. Twachtman-Reilly et al., 2008), the present review identified food selectivity as the most common feeding issue autistic youth experience. Many children without autism demonstrate food selectivity; however, such problems are typically transient and, to some extent, considered developmentally appropriate (Samuel et al., 2018). Given that most research in this area has used cross-sectional designs, factors that influence the development and maintenance of feeding and eating problems are unknown. Longitudinal research is greatly needed to improve knowledge about the comorbidity of feeding and eating symptoms with autism. It is possible that developmentally appropriate issues with feeding are amplified in youth with autism (e.g. because of sensitivity to textures), making it difficult to determine when behavior is developmentally appropriate and when more serious pathology is present. Furthermore, while the problem of inconsistent nomenclature is also present in the general population literature, the unclear boundary between feeding and eating problems further complicates the identification of such problems in youth with autism and likely puts individuals at risk for diagnostic overshadowing (i.e. where symptoms are misattributed to a person’s autism diagnosis when a co-occurring problem is present). Consistent nomenclature and definitions need to be established to ensure studies are measuring the same constructs and to begin to develop a framework to discern when feeding behaviors transition from developmentally appropriate to representing an underlying pathology. Therefore, similar to the distinction between feeding disorders and eating disorders made in this article, we propose that “feeding problems” be used to refer to eating-related behaviors and/or symptoms of feeding disorders that are unrelated to weight, shape, and/or body image concerns, yet impair functioning. Conversely, we propose that “eating problems” be reserved to describe disturbances in eating-related behaviors accompanied by preoccupation with food, eating and/or body weight or shape that impair functioning. A similar argument has been made with respect to whether ARFID best represents a feeding or eating disorder (Kennedy et al., 2018; Sharp & Stubbs, 2019). Using the term “problems” is intended to reflect the full spectrum of feeding or eating disorder symptoms rather than the term “disorder,” for which all criteria must be met. Problems with food selectivity, food neophobia, and ARFID exist at the crossroads of feeding and eating disorders (Sharp & Stubbs, 2019); however, such problems are also at the intersection of autism symptoms, emphasizing the importance of future research examining the distinction between feeding and eating problems in this population.

Another issue identified in the current review that spans feeding and eating disorders is eating rituals commonly experienced by autistic individuals. While such rituals may be related to characteristics of autism, the overlap of these problems further supports the need for more research that examines autism and eating disorders more broadly. Our finding that only three studies have examined cognitive concerns related to weight, shape, and/or body image in youth with autism highlights the paucity of research in this area. Although only a small proportion of studies included examined eating problems, results suggest that youth with autism may be at higher risk for developing disordered eating than their peers. Our results are bolstered by rapidly accumulating research suggesting a potential overlap between autism and AN in adults (e.g. Karjalainen et al., 2019; Westwood et al., 2018; Westwood & Tchanturia, 2017 provide a comprehensive review in adults). While some evidence suggests that disordered eating behaviors are present in youth with autism, research on body image in relation to disordered eating in this population is non-existent, despite body image disturbance being an important feature of AN and BN (APA, 2013). Research on body image in this population is critical to understanding the overlap between these issues and has important implications for the identification and treatment of eating disorders in this population. That is, understanding how persons with autism perceive their body and whether they endorse cognitive concerns related to weight and/or shape is essential for the differentiation of feeding disorders from eating disorders (e.g. AN from ARFID), which has important implications for treatment, as described below.

Our second major aim sought to identify characteristics of youth with autism and feeding or eating problems with a focus on cognitive functioning, adaptive functioning, and autism traits. Of the few studies that reported mean scores of cognitive functioning, there was marked variability across samples, meaning results cannot be directly compared. In addition, most participants from studies that reported cognitive ability had IQs of 70 or higher, so results may not extend to individuals with lower cognitive functioning. Similarly, a few studies included scores from measures of adaptive functioning and autism symptom severity in their analyses. Although only a handful of studies examined the relation between adaptive functioning and feeding or eating problems, the one study (Kuschner et al., 2015) that provided support for a negative relation (i.e. poor adaptive functioning skills associated with more feeding issues) had a much older sample than the others. This specific result suggests it is possible that food selectivity problems are only reflected in adaptive functioning scores as children with autism get older. While many more studies reported scores from measures of autism traits in relation to feeding or eating problems, results across studies were evenly split between evidence for and against an association. These variable findings are likely in part attributable to sample differences (e.g. differing age ranges and diagnoses such as autism spectrum disorder (ASD) vs PDD-NOS) as well as the various measures used to assess symptoms, which have differing levels of psychometric evidence.

Future research should aim to use gold-standard assessment measures (where they exist) to understand the relation between individual characteristics and feeding or eating problems in persons with autism, which would better allow for direct comparison across studies. In addition to understanding how cognitive functioning, adaptive functioning, and severity of autism traits may impact feeding and eating problems, more research is needed to understand broader differences between those who experience feeding versus eating problems. For instance, understanding whether verbal abilities, social awareness, and/or theory of mind affect the presentation or development of feeding versus eating problems, as well as clarifying if feeding problems precede eating problems in this population.

### Clinical implications

Although there is still much to understand in this area, results from this review have important implications for health-care professionals providing diagnoses and support. To identify feeding and eating problems in youth with autism, careful, nuanced investigation is critical. While diagnostic parsimony is important, a thorough evaluation must be conducted to determine whether behavior represents distinct eating or feeding pathology before attributing symptoms to a person’s autism diagnosis. An additional diagnosis may not necessarily be warranted, but gathering a comprehensive understanding of the child’s feeding or eating problems will allow health professionals to determine the most appropriate treatment. In addition, using consistent nomenclature in a patient’s electronic health record is necessary for appropriate monitoring of threshold and sub-threshold feeding and eating problems. The inclusion of such problems in a patient’s health record also provides evidence of medical complexity which has important ramifications for billing and time spent on encounters.

A recently developed, freely available measure that may prove helpful for assessment and treatment planning of feeding and eating problems is the Pica, ARFID, and Rumination Disorder Interview (PARDI; Bryant-Waugh et al., 2019). This multi-informant, semi-structured interview considers different rationales for food restriction (e.g. sensory sensitivity, lack of interest in eating, and fear of negative consequences), which can be used to guide appropriate treatment approaches. Although the PARDI has not yet been used in samples of youth with autism, it appears promising for use in this population based on the content and structure of the measure. Future research should assess the psychometric properties of the PARDI among autistic youth.

Differentiating feeding problems from disordered eating is critical for effective treatment of symptoms. Behaviorally based interventions, including cognitive behavioral techniques, have been shown to effectively treat youth with food selectivity (Dumont et al., 2019; Lukens & Silverman, 2014), whereas family-based treatment (FBT), previously known as the Maudsley family therapy, has accumulated the most evidence of efficacy for adolescents with eating disorders such as AN or BN (Lock, 2015). Limited research exists on the efficacy of treatments for youth with co-occurring autism and feeding or eating disorders; however, the presence of autism has been suggested to contribute to the resistance to conventional therapies (Wallier et al., 2009). In addition, to our knowledge, no eating disorder treatments modified for autistic youth currently exist. Regardless of the modality used, given the heterogeneity of issues autistic youth experience, treatment protocols should consider the person’s autism and eating- or feeding-specific needs to improve outcomes.

As children age, increased emphasis is placed on eating within a broader social context (e.g. eating with friends or peers; Stok et al., 2015). In light of some of the common feeding and eating problems identified within the current review (e.g. eating rituals and selective intake), some youth may require support to manage social difficulties related to their autism symptoms and their feeding or eating problems. Consequently, educators and school psychologists can play an important role in assisting youth to manage the social context of eating/feeding at school by helping mitigate related stressors. Recently, Folta et al. (2020) found that many autistic youth reported a range of strategies to cope with feeding problems in the social context, leading authors to suggest that support should involve a responsive approach that incorporates skills youth have developed to navigate eating in social situations. Finally, it is important to consider the role of caregivers in the treatment of feeding or eating problems in autistic youth. Understanding the context in which feeding or eating problems occur can help identify appropriate treatment components (e.g. psychoeducation and improving parent–child feeding relationship).

### Limitations

Although this review represents an important step toward understanding feeding and eating problems in autistic youth, it has some limitations. First, our search was limited to empirical studies that used an objective measure of feeding or eating problems, and so may have excluded qualitative studies examining this topic. While using objective measures is important to characterize behaviors, valuable information on unmeasured related issues (e.g. parental approach to feeding and parent–child feeding relationship) could not be fully understood. Relatedly, because we did not include studies exclusively examining mealtime behaviors, an understanding of the context in which the feeding and eating problems occur was limited. Second, given the variable terminology in the literature, it is possible that, despite our efforts to be inclusive, some relevant empirical studies may have been inadvertently excluded. Finally, because we excluded investigations of feeding and eating problems in the presence of another medical condition (e.g. gastrointestinal disorders and epilepsy), our results may not be representative of the full breadth of feeding and eating problems that occur in the heterogeneous population of autistic youth.

## Conclusion

Despite inconsistent terminology to describe the specific feeding and eating problems autistic youth may experience, there is clear evidence that such issues are common in this population. Most studies indicated that food selectivity was a pervasive issue among their samples of autistic youth. Only a few studies examined eating problems, but they suggested that youth with autism may be at higher risk than others for developing eating problems. The presentation and occurrence of feeding and eating problems are likely affected by factors including age, sex, gender, cognitive and adaptive functioning, and severity of autism traits. Research on feeding and eating problems in youth with autism is continually expanding, although particular attention needs to be placed on the co-occurrence of eating disorders and autism. Given that such problems affect a large proportion of autistic youth, continued research that aims to understand the prevalence, development, maintenance, and potential remission of feeding and eating problems is critical.

## Notes

1. This article uses the terms *autistic* and youth *with autism* interchangeably, to acknowledge and respect both person-first and identity-first language.
